# Biotechnology Applications of Cell-Free Expression Systems

**DOI:** 10.3390/life11121367

**Published:** 2021-12-08

**Authors:** August Brookwell, Javin P. Oza, Filippo Caschera

**Affiliations:** 1 Department of Chemistry & Biochemistry, College of Science & Mathematics, California Polytechnic State University, San Luis Obispo, CA 93407, USA; abrookwe@calpoly.edu; 2 Nuclera Nucleics Ltd., Cambridge CB4 0GD, UK

**Keywords:** cell-free expression systems, cell-free protein synthesis, biotechnology applications, synthetic biology, metabolic engineering, prototyping, biomanufacturing, machine learning

## Abstract

Cell-free systems are a rapidly expanding platform technology with an important role in the engineering of biological systems. The key advantages that drive their broad adoption are increased efficiency, versatility, and low cost compared to in vivo systems. Traditionally, in vivo platforms have been used to synthesize novel and industrially relevant proteins and serve as a testbed for prototyping numerous biotechnologies such as genetic circuits and biosensors. Although in vivo platforms currently have many applications within biotechnology, they are hindered by time-constraining growth cycles, homeostatic considerations, and limited adaptability in production. Conversely, cell-free platforms are not hindered by constraints for supporting life and are therefore highly adaptable to a broad range of production and testing schemes. The advantages of cell-free platforms are being leveraged more commonly by the biotechnology community, and cell-free applications are expected to grow exponentially in the next decade. In this study, new and emerging applications of cell-free platforms, with a specific focus on cell-free protein synthesis (CFPS), will be examined. The current and near-future role of CFPS within metabolic engineering, prototyping, and biomanufacturing will be investigated as well as how the integration of machine learning is beneficial to these applications.

## 1. Introduction

**Figure 1:**
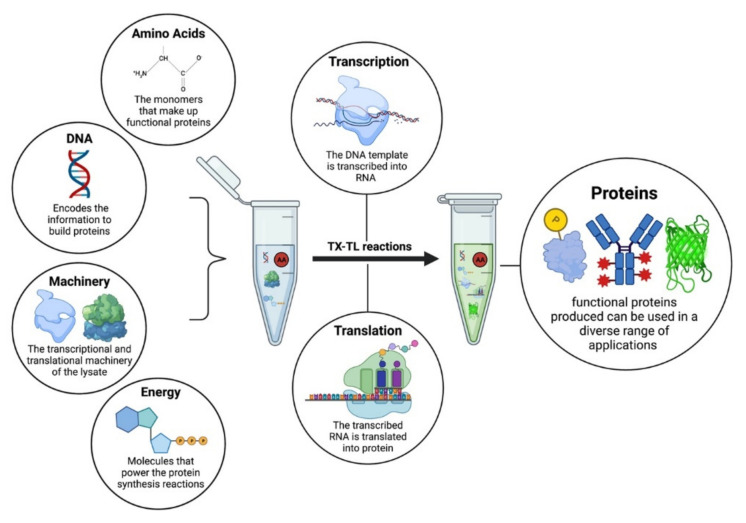
The components of a cell-free protein synthesis reaction. The reaction is assembled in a test tube, i.e., DNA, amino acids, and energy buffers are mixed along with the molecular machinery present in the cellular lysate to initiate transcription and translation for the synthesis of functional proteins.

Cell-free systems can generally be defined as platforms where biochemical reactions occur independently of living cells. Cell-free systems are divided into two types based on the method of preparation: extract-based systems and enzyme-based systems [1]. The extract-based cell-free systems were first introduced over a century ago by Eduard Buchner. Buchner demonstrated that cell extracts prepared from yeast could ferment sugar independent of the living yeast cells themselves [2], a discovery that earned him the 1907 Nobel Prize in Chemistry [3]. Decades later, in the mid-20th century, molecular biologists used cell extracts to study the dynamics of protein synthesis in prokaryotic and eukaryotic organisms [4]. The first application of cell-free protein synthesis (CFPS) was seen in a study of liver cell extracts from rats [5], and soon after, researchers began making extracts from a variety of other organisms to study protein synthesis in a cell-free format [6,7,8]. Early CFPS systems were based on “S30 extracts”, supernatants prepared from numerous different cell types through centrifugation of their lysates, with “S30” denoting supernatants centrifuged at 30,000× *g*. Over the subsequent decades, researchers used S30 extracts as a testbed to improve CFPS by adding different polymerases and other enzymes. Figure 1 shows the cell-free protein synthesis components.

Iterative improvements have allowed for the application of CFPS for broader research objectives, such as observing gene regulation through the coupling of transcriptional and translational machinery [9] and the ability to run extended synthesis experiments in cell-free systems by adding features such as dialysis membranes that maintain a continuous flow of resources for synthesis reactions [10]. Further optimization of cell-free extracts included drawing from lysates of bacterial strains with specific advantages such as those that lack nucleic acid degrading enzymes [9,11] or from extremophiles with high-temperature tolerances [12]. In the 21st century, researchers have turned to further mirroring cellular conditions as a method of improving and prolonging protein synthesis. Some examples include genetically engineering bacterial strains to improve the metabolic conditions associated with growing amino acid chains [13] and the utilization of metabolic pathways within extracts, which greatly improve synthesis durations in batch formats [14]. In contrast to extract-based systems, enzyme-based cell-free systems are prepared by mixing purified enzymes to further define the cell-free milieu and improve outcomes for CFPS. Platforms can be tailored to contain only factors that would be beneficial to protein production and not those that are potentially detrimental, including degradation enzymes and energetically costly pathways not tied to synthesis, which are present in extract-based systems [15].

**Figure 2:**
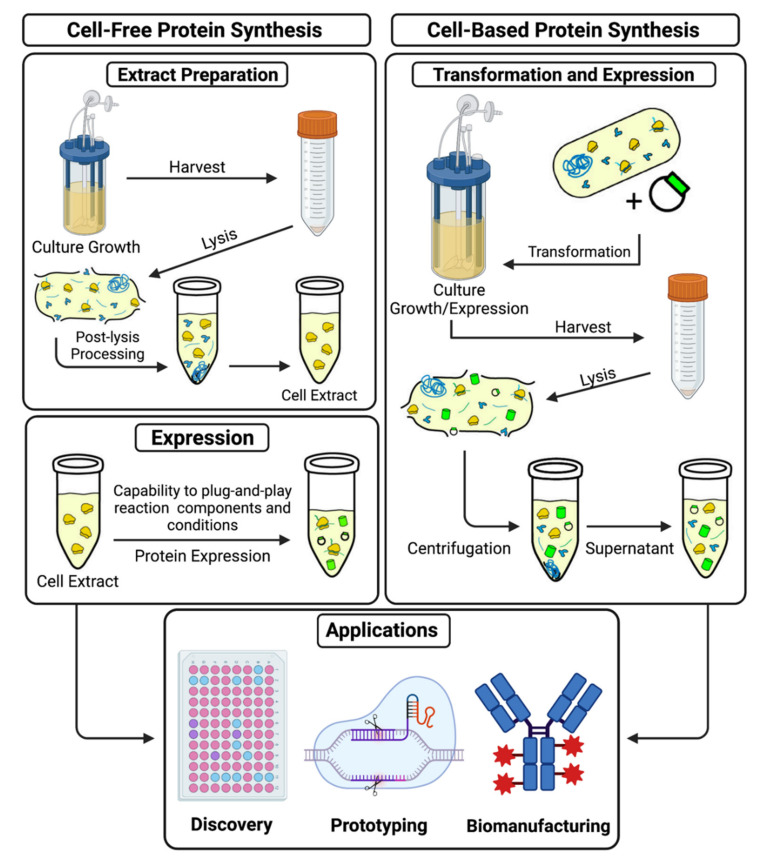
Cell-free and cell-based protein synthesis systems. The figure illustrates a comparison between extract preparation for in vitro protein synthesis and the procedure for in vivo protein synthesis.

Traditionally, in vivo systems were the preferred platform for protein production because of the technological limitations of CFPS, namely short synthesis duration, resource depletion, low yields, and difficulty in scalability compared to protein production in vivo [16]. With recent optimization and standardization of cell-free systems, the potential scope of applications where CFPS systems are more advantageous has broadened significantly [17]. Living cells are highly complex and require specific conditions to maintain proper homeostasis. The complexity makes controlling reactions occurring within the cell membrane difficult and incompatible with modular modifications [18]. Cell-free systems are not bound by these same homeostatic considerations because they contain no living cells. This means that all the energy of the system is dedicated to the singular goal of producing a target molecule, rather than being divided between multiple cellular processes working to keep the cell alive and healthy [19]. Cell-free systems can also be tailored to produce a broad range of different target molecules simply by swapping out components best suited to produce those molecules, such as particular lysates or polymerases. The open environment also allows for the observation and alteration of the real-time protein synthesis reaction [19,20]. Figure 2 shows the comparison between cell-free and cell-based protein synthesis.

The open environment features make cell-free platforms particularly advantageous for the prototyping of new metabolic pathways and genetic circuits, where parameters can be more easily controlled without the confounding variables found in in vivo systems [21]. Cell-free platforms also benefit biosensing efforts, allowing for both the addition of modules that increase sensitivity and response-time of biosensors, as well as the real-time observation of these modifications in vitro [22]. Cell-free systems are safer for environmental biosensing and bioremediation efforts, while competing in vivo solutions involve the potentially dangerous and controversial aspect of releasing genetically modified organisms into sensitive environments [22,23].

Biomanufacturing is another industry that stands to benefit from cell-free biotechnology, especially in the on-demand production of target proteins. Historically, cell-based systems have had the edge over cell-free systems in the scalability of biomolecule production, where the previously mentioned technological limitations of cell-free systems prevented their use on an industrial scale [16]. Recent demonstrations of large volume CFPS reactions [24] and the aforementioned optimization of cell-free platforms addressing former technological hurdles have revitalized interest in these platforms for biomanufacturing [16]. Cell-free platforms also have numerous advantages over cell-based platforms on an industrial scale, such as faster development times, adaptability to various production schemes, resistance to conditions toxic to cell-based systems, and cost-effectiveness at scale [25]. The increasing demand for low-cost and sustainable products manufactured from low-carbon emission methods can also be addressed using cell-free systems optimized to utilize more sustainable carbon utilization pathways and green energy regeneration modules [20,25,26]. While cell-free platforms have been demonstrated to be adaptable to industrial production schemes, they can also be utilized for smaller-scale on-demand production of target molecules. This is particularly useful for the on-demand production of therapeutics, of which many niche drugs do not have the demand for pharmaceutical companies to warrant building expensive cell-based infrastructure to produce them [26]. Cell-free systems could be used at a scale where the on-demand manufacturing of custom therapeutics for individual customers would be feasible [27].

Although cell-free systems have been demonstrated to have many advantages over cell-based systems, these systems can still be further optimized. One pathway towards further optimization is automation. Cell-free platforms can be optimized to use microfluidic volumes, and their standardized protocols can be adapted to automated use [28]. Cell-free platforms can also benefit from machine learning algorithms, which can significantly assist in optimizing the platform for more efficient prototyping and high-throughput experimentation [29]. Combining automation and machine learning optimization of cell-free systems has even been demonstrated to improve protein production yields, and the same optimization could be used to improve the prototyping of genetic circuits and metabolic pathways [30]. The trend towards the integration of automation in manufacturing, generally coupled with the ease of integration with cell-free systems, represents another significant advantage of cell-free platforms over cell-based systems.

The applications of CFPS and other cell-free systems are growing as the advantages of these platforms become more apparent. Driving this push towards cell-free systems are innovators optimizing these platforms to fill many different roles, from industrial-size protein production factories to miniaturized drug discovery instruments. The optimization of cell-free platforms for these various roles showcases the adaptability and versatility of the platform, and as a result, demonstrates its growing use over cell-based systems. This study will review the emerging and future applications of cell-free systems. The utility of cell-free platforms over cell-based platforms, especially as they pertain to CFPS, will be examined in the context of prototyping, metabolic engineering, and biomanufacturing, as well as how automation and machine learning will further optimize cell-free systems. Current roadblocks to the successful implementation of cell-free systems in these applications will be addressed, providing a context for what challenges lay ahead for the next generation of cell-free researchers.

## 2. Cell-Free Metabolic Engineering

Metabolic engineering is the manipulation of a cell’s genetic and molecular processes to gain insights and control over biological functions such as enzymatic pathways, signal transduction, and gene expression. Metabolic engineering efforts have utilized cell-free systems since the field’s inception. The first example of cell-free metabolic engineering (CFME) was in the early 1960s, when Nirenberg and Matthaei demonstrated the cell-free protein synthesis of a single amino acid via a simple polypeptide synthesis system [31]. The system was used less than a decade after Watson and Crick successfully determined the molecular structure of DNA, and researchers were hard at work determining what would later become known as the “central dogma”, the expression of genes into functional proteins via transcription and subsequent translation [32,33]. Research into the genetic code led to the creation of many fields within the fledgling molecular biology community, including what would eventually be known as metabolic engineering.

Despite continued research into the nuances of protein synthesis, for years, the process was not well understood by researchers [34]. CFME experiments helped change this by revealing aspects of protein synthesis in a compartmentalized manner that allowed researchers to build a complete picture of the synthesis process. CFME experiments continued throughout the next few decades, with researchers improving the duration of protein synthesis and the ability to synthesize functional products as they began to elucidate the molecular factors of protein synthesis and the surrounding cellular processes [34]. The next significant breakthrough in CFME came in the late 1980s with the demonstration of the continuous-exchange cell-free system [10]. The continuous-exchange cell-free system was able to produce viral coat proteins for 20 h and calcitonin polypeptides for 40 h continuously using both prokaryotic and eukaryotic platforms [10]. The system proved that the previous roadblocks that limited the effectiveness of cell-free systems of the past, namely resource exhaustion and short protein synthesis durations, could be overcome via a continuous system [34]. Despite this breakthrough, systems being utilized for CFME still suffered from significant drawbacks, including low yields of target proteins and primitive conversion of single enzymes and metabolites to regenerate ATP and GTP [34]. Researchers also had to contend with the cost-prohibitive nature of the energy reagents used to power CFME reactions. Producing a single gram of protein product, the cost in energy reagents would be ~$30,000 at 10 mg/h [34].

Similar to how the introduction of continuous-exchange cell-free systems removed previous roadblocks in CFPS, new CFME experiments that altered the cell-free platform metabolically allowed for new possibilities for metabolic engineering applications in cell-free. One of these experiments demonstrated that adding cofactors into the reaction mix that inhibit or activate enzymes and pathways that directly interact with the protein synthesis reaction can improve yields and synthesis duration. The cofactors included: increasing the amount of ATP produced in reaction by adding additional factors to react with pyruvate; increasing the concentration of amino acids in the reaction mix to prevent resource exhaustion; and the inhibition of the futile cycle of PEP synthetase via oxalate addition which prevented conversion of phosphoenolpyruvate product back into pyruvate precursor [35]. Another experiment showed an enzyme-based cell-free platform that exhibited CFPS reactions in two separate formats: using purified components rather than a cell lysate and coupled transcription/translation [15]. The experiments legitimized methods for optimizing cell-free that are used two decades later to perform CFME experiments and proved that cell-free platforms capable of competing with cell-based systems were attainable [34]. More recently, new advancements in the metabolic engineering of cell-free systems have led to further optimization of lysates and greater control over enzymatic pathways. The Jewett Lab at Northwestern University has demonstrated the overexpression of enzymes prior to lysis and the subsequent mixing of overexpressed enzymes within a single lysate [36]. Mixing allows for a greater degree of customization in lysate-based cell-free systems, allowing for a broader range of expression and the ability to control whole pathways via CFPS [36].

CFME today is much more industrially relevant than at any other time in the field’s history. Cell-free platforms are involved in the production of next-generation pharmaceuticals, food products, cosmetics, and other industrially relevant biomolecules and are sanctioned by the FDA [34]. The ability to scale production, control the reaction factors, and produce a wide range of target proteins via metabolic tuning characterizes the advantage CFPS and CFME have over cell-based systems [34].

## 3. Comparing Extract and Enzyme-Based CFME

When comparing extract-based and enzyme-based CFME platforms, each has advantages, disadvantages, and unique considerations and is, therefore, more suited to specific reaction schemes [34,37]. Extract-based systems as they are known today began to take shape in the 1990s and centered around crude cell extracts for batch reactions, which are extracts consisting of crude lysates of cells that contain many cellular enzymes and cofactors absent purification [34]. Crude cell-free extracts are advantageous compared to traditional fermentation setups because, in a cell-free format, the entire batch is a single reaction mix that can be assessed and controlled in real-time. In contrast, standard fermentation consists of isolated bioreactors (cells) suspended in solutions that are much harder to manipulate [34]. This is also the case when comparing enzyme-based systems to fermentation setups; however, the comparison better applies to extract-based systems because of the latter’s ability to scale to industrial production levels [37]. The chief similarity between both platforms is energy expenditure. The translation is the most energetically costly part of the synthesis reaction, costing ~40 mM of ATP to synthesize 1 mg/mL of a 25 kDa protein in batch format [38]. For this reason, continuous formats that recycle energy may be advantageous for both systems depending on the production scheme.

Enzyme-based systems have the advantage of total reaction parameter control, limiting the possibility of competing reactions and toxic cofactors that hinder extract-based systems [37]. The most well-documented example of an enzyme-based platform is the PURE (Protein Expression Using Recombinant Elements) system, the most complex purified component system currently devised, with 82 isolated macromolecules, of which 46 are tRNAs, making up its reaction setup [15]. The PURE system, and similar purified component platforms, are especially adept at translating otherwise difficult-to-express proteins, such as insulin, which is a challenge even for eukaryotic organisms such as yeast [39]. This is due to the fact that reaction setups in enzyme-based systems can be easily modified and do not have to rely on nonmodel organisms or their lysates to produce complex products. Enzyme-based systems are not without their disadvantages, however, which manifest mostly in scalability issues [37]. Currently, purified components for enzyme-based systems are costly, and scaling these platforms to industrial production levels is currently cost-prohibitive [37]. In response, industry startup companies such as FabricNano are devising new ways of lowering the cost of these purified components. One method currently being researched is the immobilization of biocatalysts such as enzymes and their cofactors for continuous flow systems [40]. Immobilizing enzyme cofactors as well as the enzymes themselves allows them to recycle themselves in solution, removing the need to replenish these biocatalysts and significantly lowering costs [40]. The recycling is accomplished via a swinging motion of the enzyme-substrate, where the substrate is covalently linked to the enzyme and can “swing” between the many active sites present on the enzyme surface [40]. The mechanism prevents the substrate from diffusing by allowing for its continual reuse on the same enzyme [40]. Although technologies such as this are being developed to lower the cost of utilizing purified components, these methods are new and have yet to be broadly implemented. It is for this reason that extract-based systems are used for industrial production applications.

Extract-based systems are more advantageous for scaling to industrial levels than enzyme-based systems [37]. In the last 20 years, numerous improvements in energy regeneration for batch formats have increased reaction duration and potential product yields [37,41,42]. With these improvements, yields in batch formats have been reported to reach 2.34 mg/mL [43] and 6 mg/mL in continuous formats [42]. Recently, a system update based on the coupling of maltodextrin and d-ribose with a high-energy phosphate donor synthesized 4 mg/mL of a fluorescent reporter in batch format [44]. Despite these improvements, batch formats still suffer from side reactions that limit energy efficiency [37]. The use of low-cost energy sources such as polyphosphate and maltodextrin [45], glucose, and other monosaccharides can mitigate the detrimental effects of these side reactions, and other techniques such as harvesting cell extract at peak translation and removing endogenous nucleic acids from the extract can be employed to further improve efficiency [37].

## 4. Cell-Free Prototyping

Before biological and biochemical systems can be optimized for industrial applications such as mass production, these systems must first be proven in the prototyping stage. Prototyping is an experimental process where a novel idea, tool, or system is tested. In the context of biotechnology, this can apply to drug trials, metabolic pathway discovery, genetic circuit testing, and a host of other prototyping applications. Each of these examples requires a platform or an environment for validation. The platforms can be biological in nature, such as recoded *E. coli* strains, or biochemical, such as cell-free lysates or purified component cell-free systems. Cell-based systems have traditionally been utilized for a diverse range of different prototyping endeavors. In the past, these systems had significant advantages over cell-free systems in the prototyping of these platforms for industrial applications because of technological limitations of cell-free systems in scalability and process duration [16]. However, improvements in cell-free systems in the last decade, such as the engineering of high-yield CFPS systems, have changed this paradigm [17,24]. Today, cell-free systems have numerous advantages for prototyping over cell-based systems. Using cell-free platforms with coupled transcription/translation systems, research into cell metabolism can occur independently of potential conflicting reactions and confounding variables [37]. This is an inherent advantage of working with a cell-free system because researchers control all experimental parameters, confining the biochemical reactions that occur within a test tube to the experiment designers’ choosing [37]. Another important factor in prototyping for industrial processes, for instance, is reaction conditions. Cell-based systems, especially those utilizing model organisms optimized for biomolecule production, are extremely limited in their tolerances of temperature, salt concentration, pH, and toxic conditions [46]. Utilizing nonmodel organisms with special tolerances to these conditions can broaden the usefulness of cell-based systems to a degree, however many of these organisms’ optimal conditions for industrial applications are not well elucidated, and maintaining homeostasis is still a requirement that limits useable energy for target synthesis reactions [19,46,47]. By contrast, cell-free systems’ ability to be optimized to a diverse range of environmental stressors stands as one of its primary advantages over cell-based systems in prototyping [46]. For example, the PURE system, the first demonstrated synthetic cell-free system built via purified components rather than cell lysate, has been able to synthesize a number of difficult-to-express proteins because of its adaptability to different reaction setups [15]. This adaptability is also useful in prototyping drug candidates, where researchers can not only measure the drug action to a specific pathway independent of competing cellular processes but also expand the repertoire of possible therapeutic molecules that can be synthesized [48]. This advantage is especially important when considering broader natural product discovery since most natural molecules with potential applications as pharmaceuticals and other industrially relevant applications have yet to be discovered [49]. Cell-free platforms, with a greater range of reaction tolerances and adaptability to different production schemes, are ideal candidates for this product discovery prototyping. Another important advantage cell-free platforms have in the prototyping space is shortened timescales from reaction start to results [37]. What takes cell-based systems days or potentially weeks to complete, cell-free systems can complete in hours [37]. This is a huge advantage in prototyping systems, such as genetic circuits, because this rapid turnaround time allows for the rapid design to debugging cycles [37]. A prime example of this is the Noireaux Lab’s utilization of a coupled transcription/translation cell-free system to enable research into the applications of CRISPR [50]. Clustered Regularly Interspaced Short Palindromic Repeats (CRISPR) is a gene-editing system acquired from a prokaryotic defense system where a collection of DNA sequences acquired by prokaryotes from former bacteriophage invaders provide a defense against viruses with similar DNA segments [51]. Since the CRISPR system’s discovery, numerous enzymes and other biomolecules have been found that have the potential to optimize the system’s functionality [50]. Cell-based validation of these CRISPR factors, as well as in vitro assay methods of validation, suffer from the slow turnaround times associated with either culturing live cells or purifying the relevant proteins [50]. By using a cell-free system to work around these issues, the Noireaux Lab was able to rapidly characterize a wide range of CRISPR-relevant biomolecules, such as nucleases and gRNAs [50]. Cell-free similarly benefits other prototyping pursuits such as drug discovery, decreasing the time from discovery to validation. Compounding these advantages is the ability to monitor cell-free reactions in real-time, which greatly benefits prototyping efforts by elucidating the mechanisms underlying the observed reactions [37]. Another factor increasing the speed of cell-free prototyping is linear DNA. The use of linear DNA expression to prototype genetic circuits is alluring because it could drastically reduce the time spent on prototyping and validation cycles, limiting the need to transform sets of plasmids in vivo [52]. Traditionally, using plasmid DNA for genetic circuit prototyping is time-consuming, taking multiple days per cycle to validate. Linear DNA can run the same cycles in only 4-8 h, allowing for the validation of large genetic circuits, bypassing validation in vivo, and possibly allowing for new studies into molecules previously deemed too toxic to work with [52].

## 5. Cell-Free Biosensing

Biosensing is the detection of biochemical and chemical signatures in a system using a biological platform. Biosensors utilize a diverse range of bioreceptors such as enzymes, antibodies, or nucleic acids, coupled with what is known as a transducer. Transducers convert energy from interactions with the target analyte to a signal proportional to the concentration of the analyte [23,53]. Cell-based biosensors take advantage of host metabolic pathways or utilize genetic modification to detect target analytes, however, their use is problematic for a variety of reasons [22,23]. Firstly, operating within the cell membrane is fraught with challenges, such as difficulty modifying the biosensor parameters within the cell membrane. Secondly, genetic modification of a cell is time-consuming and is potentially environmentally damaging or dangerous [23]. Testing for specific analytes in nature using cell-based systems requires releasing those genetically modified organisms into the environment, which could adversely affect an ecosystem and is ethically controversial [23]. Thirdly, cell-based biosensors have a limit on the analytes that can be engineered to detect and the specificity with which they can detect those analytes. This is because cells have homeostatic concerns and, thus, cannot operate in certain environments and are limited in detecting toxic analytes at high concentrations [23]. Lastly, cell-based systems can undergo evolutionary changes that can erase a previously established function of the biosensor, leaving the ability to detect an analyte diminished or entirely removed [23]. Cell-free systems circumvent these concerns by operating without the need to keep organisms alive. Without cell membranes, cell-free systems operate within a homogeneous lysate that can be more easily monitored and modified. Also, cell-free systems do not risk the release of genetically modified organisms into nature, as simple metabolic pathways do not pose the same environmental threat as engineered microbes [23,54]. Without homeostatic concerns, cell-free biosensors can also operate in a diverse range of environments that would otherwise be toxic for cell-based systems, and cell-free systems can achieve higher sensitivities to toxic analytes because of the systems’ ability to withstand higher concentrations of toxins [23,54]. Finally, cell-free systems are also not subject to concerns about evolutionary change altering sensor function [23].

In the past, the high cost of cell-free reagents prohibited their use in biosensing. However, technological innovations in cell-free have lowered the cost of preparing extracts that can be optimized for sensing applications [23,55]. Cell-free biosensors can be optimized to detect a range of possible analytes, and multiple biosensing strategies are compatible with a cell-free platform [23]. One possible avenue of detection is the utilization of transcription factors for the detection of target ligands. Transcription factor detection involves the expression of a reporter molecule such as green fluorescent protein (GFP) in the presence of a target analyte, where the presence of the analyte will cause an inhibitory molecule, which is bound to the operator controlling the expression of the reporter molecule, to break off, allowing for the expression of the reporter [54,56,57]. Another possible detection method utilizes riboswitches, which are RNA structures that regulate gene expression through the binding of certain metabolites [23]. In the presence of a minimum concentration of a target analyte, Riboswitches will change conformation, and a change in expression can be measured [56]. Other detection methods utilizing DNA aptamers or quorum sensing have also been engineered in cell-free systems [23].

Along with innovations in detection methods, technological revolutions in biosensor hardware have also expanded their detection abilities and overall use. Nowhere is this innovation more prevalent than in the wearable technology market. The wearable technology market was worth over 30 billion dollars in 2019, with a CAGR of 15.9% [58]. This growth in wearable technology and biosensors is due in large part to improvements in hardware materials, as well as the integration of new technologies such as wireless communication [59]. Wearable technology represents an ideal platform for a variety of different detection methods. However, cell-based systems have not seen integration into wearable platforms because of the limitations in keeping cells alive on time spans comparable to other detection methods [59]. However, research has been performed on integrating cell-free systems into wearable biosensors to detect potential pathogen exposure and overall health monitoring [59]. Cell-free wearable biosensors utilize freeze-dried, cell-free reactions (FDCF), a technology that has already seen use as a standalone biomolecule diagnostic tool and in educational kits [60,61,62]. FDCF systems demonstrated cell-free wearable biosensors and utilized CRISPR technology to detect target analytes such as nucleic acids [59]. The biosensor can also be optimized to use other genetically engineered systems, such as riboswitches and aptamers, to broaden the detection of target pathogens and toxins [59]. Perhaps the most relevant wearable application of the cell-free biosensor is a face mask with an integrated FDCF CRISPR biosensor capable of SARS-CoV-2 detection [59]. Cell-free biosensors that can operate free of any device are also attractive for applications where factors such as weight and portability are especially important. One such example of this is the utilization of the PURE system towards biosensing, which demonstrated compatibility with colorimetric reporter enzymes that would allow simple visual detection of multiple target analytes [63]. Technological innovations in the integration of cell-free systems demonstrate the advantage they present for the biosensing of target analytes, especially those that are toxic or present challenges for cell-based systems.

## 6. Cell-Free Biomanufacturing

Biomanufacturing is the process by which chemicals and materials of commercial value are produced via biological, biochemical, and chemical synthesis platforms. The first examples of biomanufacturing date back thousands of years to the dawn of human civilization in what is today modern Iraq, where records indicate the production of alcoholic beverages via fermentation [64]. Various other ancient cultures have been recorded engaging in the biomanufacturing of beer, wine, cheeses, and other food items, utilizing solid-state fermentation in mixed microorganism cultures [64,65,66]. In modern history, three main technological revolutions characterize advancements in biomanufacturing. The first occurred in the first decade of the 20th century and centered around the production of simple metabolites via monoculture microorganisms and large-scale anaerobic liquid fermentation [64]. The metabolites had intrinsic functions in the organisms they were produced from and included various alcohols, ketones, organic acids, and amino acids [64]. Driving this innovation in biomanufacturing and biomolecule discovery were the technological and economic booms occurring in the early 20th century, which drove the creation of synthetic rubber, solvents, paints, resins, etc. [64]. The second revolution in biomanufacturing centered around the production of pharmaceuticals, such as penicillin and other secondary metabolites, utilizing mutated fungi and bacteria as well as aerobic submerged fermentation [64]. Research into optimal media, growth conditions, and physiological control of cultures, characterized this period of antibiotic discovery as the beginnings of biochemical engineering [67]. The third revolution in biomanufacturing centered around recombinant protein and enzyme production via advanced cell cultures and recombinant DNA technology [64]. Development of this technology allowed for the production of next-generation therapeutics such as monoclonal antibodies and small molecules for cancer treatments. Much of this progress was driven by the plateauing of chemical synthesis platforms unable to produce complex therapeutics cost-effectively. Cell-based systems were able to meet this challenge, with mammalian cell cultures demonstrating low-cost production of complex biomolecules, such as glycosylated proteins [68]. Beyond therapeutic targets, bioproduction of proteins such as polymerases and restriction enzymes for academic research was made possible by cell-based systems, and industrial production of enzymes via optimized fermentation methods was perfected [64].

Presently, modern and emerging methods of production constitute the fourth technological revolution in biomanufacturing. Regenerative medicines, metabolic engineering, synthetic biology, and other emerging fields are already impacting the industry [64]. Companies leading this technological revolution include LenioBio, a biotechnology company that has developed ALiCE^®^, a scalable cell-free eukaryotic expression platform optimized for pharmaceutical and technical protein production [69]; SwiftScale Biologics, a biotechnology company that has developed a CFPS platform that boasts g/L protein titers in single-day timescales [70]; Kykeon Biotech, a biotechnology startup that offers cost-effective and scalable cell-free production of customizable proteins [71]; and CellFree Sciences, a biotechnology company offering CFPS services for proteins derived from eukaryotic, prokaryotic, and viral hosts [72]. Biomanufacturing accounts for a market value of 200 billion dollars [73], and next-generation biomanufacturing of products such as biopharmaceuticals is expected to grow by over 8% before 2025 [74]. The projected increase in the value of the biomanufacturing industry represents an opportunity to transition to cell-free platforms of production. Cell-free platforms have numerous advantages over cell-based systems currently being utilized for biomanufacturing, including increased control over reaction parameters such as chemistry and temperature, which would, in cell-based systems, pose a risk to homeostasis [73]. They also increase monitoring of specific production processes in real-time [73], increase capability for both small-scale and large-scale biomanufacturing using the same reagents [73], increase stable storage capability via lyophilization of reagents involved in production [73], and operate in more rapid production cycles of biomolecules than cell-based systems [73]. The advantages demonstrate that cell-free biomanufacturing platforms represent a logical technological progression to a more efficient and cost-effective method of producing pharmaceuticals, food products, and other industrially relevant biomolecules.

### 6.1. Pharmaceuticals

The largest and fastest-growing of these biomanufacturing fields is the production of pharmaceuticals. The global pharmaceutical industry accounts for ~1.1–1.4 trillion dollars in market value and has had a compounded annual growth rate (CAGR) of 5.8% reported in 2017 [75]. As a subset, biopharmaceuticals are reported to account for ~40% of this global market value [76]. The growth is largely driven by global prescription drug sales, with the top 10 prescription drugs’ combined sales reported at over 355 billion dollars in 2016 [77]. The projected growth in pharmaceutical manufacturing represents an important opportunity for cell-free systems to begin producing pharmaceuticals inexpensively and more efficiently than current methods. Production of pharmaceuticals encompasses a diverse range of different molecules, including biologics such as monoclonal antibodies, vaccines, and other therapeutic proteins, as well as small molecule therapeutics that target molecular processes within cells.

#### 6.1.1. Monoclonal Antibodies

Monoclonal antibodies (MAbs) are one of the most powerful biologics available today, able to treat a variety of different disease types such as cancer and viral diseases [78]. MAbs mechanism of action varies based on their intended use; however, they are usually involved in blocking the binding of disease-causing molecules [78]. This includes binding to antigens necessary for disease processes, such as blocking viruses by binding a membrane protein on a host cell necessary for viral entry or binding to proinflammatory cytokines to limit cancer growth [78]. The research and development of MAbs are increasing with dozens of different antibody-drug conjugates (ADCs)—MAbs combined with small molecules specialized in fighting cancerous tumor cells—currently in clinical trials [79]. ADCs have significant advantages over traditional cancer treatments such as chemotherapy. Chemotherapy works by injecting a cocktail of potent chemicals into a patient with the intent of killing cancerous cells. Treatment is effective but comes at the cost of indiscriminate poisoning of both cancer and normal body cells [80]. ADCs avoid this pitfall by only targeting antigens associated with cancer cells and not the healthy cells of the patient [81]. ADCs are expected to see increased use in treating blood cancers such as leukemia and lymphoma as well as breast cancer in the next five years, also expanding into use in ovarian, lung, and brain cancers to a lesser degree [81]. Currently, most MAbs are produced via the transformation of the genes that encode the desired antibody into cell-based platforms optimized to produce that antibody [82]. The majority of ADCs, and MAbs generally, are produced in Chinese hamster ovary (CHO) cells, which involve cloning and growth cycle steps that can be time-consuming and result in limited product yields [83]. By comparison, utilizing CFPS for the production of antibodies resulted in greater yields than CHO-based production, at 0.55 g of MAb/L/day for cell-free production compared with 0.017–0.25 g of MAb/L/day for CHO-based production, depending on the cell-based method used [83]. The increasing popularity of MAb treatments and the increase in research and development of new ADC candidates coupled with the demand to produce these molecules efficiently introduces an opportunity for cell-free platforms to begin manufacturing MAbs. Cell-free platforms have many advantages over cell-based systems in the manufacturing of MAbs, including decreased manufacturing costs and faster production times [84]. Cell-free platforms have also demonstrated the capability to produce a variety of other MAbs [84,85,86]. Sutro Biopharma is one biotechnology company currently working on integrating CFPS into ADC production, with two ADCs developed through their CFPS platform XpressCF^®^ currently undergoing clinical trials [87]. The main limitation to cell-free production of MAbs is the high cost of reagents for CFPS reactions, namely T7-RNAP and reagents involving plasmid DNA [83]. However, recycling these components could bring reaction costs down significantly, decreasing the total cost by as much as 29% [83]. With new innovations in the cost-effectiveness of running CFPS reactions, cell-free platforms will be increasingly used in the production of antibodies.

#### 6.1.2. Antimicrobial Peptides

Antimicrobial peptides (AMPs) are a type of short-chain polypeptides that are involved in the innate immune response of many different prokaryotic and eukaryotic organisms [88]. AMP mechanisms are greatly dependent on the organism they are produced from, and their diverse array of functions include disruption of cell membrane stability and the inhibition of molecular processes such as protein synthesis and enzyme function [88,89]. Other functions of AMPs include the modulation of cellular apoptosis, promotion of angiogenesis, and the stimulus of chemokine production [89]. AMPs are incredibly diverse in their structure, function, and targets, thus, many different methods are used to categorize them [88]. A useful method of peptide categorization is based on activity, which focuses on the type of organism the peptide functions against [88]. The categories include antibacterial, antifungal, antiparasitic, and anticancer peptides [88]. The diverse range of peptide target categories shows the potential that AMPs have as targeted therapeutics. Other factors driving the development of AMPs include antibiotic resistance [88]—the adaptation of many microorganisms such that antibiotics that once were effective in killing a specific type of microorganism are now ineffective towards those same organisms [90]. The rising problem of antibiotic resistance is due to a variety of factors, including overuse of popular antibiotics such as penicillin, incorrect prescription of antibiotics resulting in more resilient bacterial strains, and the slow development of new antibiotics [90]. One potential solution to this problem is to utilize alternative therapeutics such as antimicrobial peptides to target biological agents [88]. AMPs are prime candidates to combat antibiotic resistance because, unlike antibiotics which have a single target that they bind to with high affinity, AMPs promote a number of antimicrobial processes simultaneously [91]. Multiple antimicrobial functions occurring in parallel have proven to be more challenging for pathogens to develop a resistance against than antibiotics [91]. This is because single-target antibiotics are much easier to develop new mutations against or propagate existing resistant mutants within a culture compared to multitarget microbial peptides [91]. Due to their small size and diverse therapeutic targets, AMPs are a prime target for cell-free expression platforms. Cell-free platforms are adept at producing proteins and other amino acid chain-related biomolecules such as polypeptides [91]. Cell-free systems have also shown the ability to produce AMPs. AMPs-like human β-defensin-2, which demonstrates antimicrobial activity against Gram-negative bacteria, and magainin 2, which displays antimicrobial activity against a broad spectrum of bacteria, have been produced in aqueous and lyophilized formats [92,93]. Cell-free systems could be optimized to produce AMPs on larger scales, and they may be especially useful for difficult-to-express or particularly large AMPs [94].

#### 6.1.3. Vaccines

Vaccines encompass a wide range of different therapeutic agents, from weakened forms of viruses to mRNA transcripts encoding viral proteins, all with the goal of acting as an antigen to activate the production of antibodies against a given disease [95]. Vaccines provide protection against a variety of primarily viral and bacterial diseases depending on the target disease being immunized against. With the recent SARS-CoV-2 pandemic affecting populations worldwide, the focus on immunization and vaccine production has increased significantly [96]. Development of facilities needed to produce mRNA therapeutics in massive quantities has been and is currently underway, with infrastructure capable of producing tens of millions of these products for vaccination and related applications [96]. The market value of vaccine production is also predicted to increase from 28 billion dollars reported in 2017 to over 80 billion dollars by 2027, with a CAGR of 8.7% [97]. With this increase in vaccine production worldwide, an updated view of vaccine manufacturing methods is required.

Currently, most vaccines are manufactured using cell-based systems such as various bacterial, yeast, and chicken eggs depending on the vaccine type [98]. Pathogens and cells harboring propagating viruses are grown in bioreactors where the target antigen will then be purified and extracted [98]. Given the expensive and time-consuming production process associated with vaccines, vaccine production could benefit greatly from cell-free production. Cell-free systems have demonstrated the capability of producing conjugate vaccines via the in vitro bioconjugate vaccine expression (iVAX) system, a platform utilizing *E. coli* lysates to generate bioconjugate vaccine doses on demand [99]. Vaccines consisting of proteins, virus-like particles (VLPs), small molecules, and nucleic acids could conceivably also be produced in cell-free systems given that this platform has successfully made nontherapeutic versions of these same types of molecules. In particular, mRNA vaccines have garnered much attention since their debut in immunizing against the COVID-19 pandemic [100]. The vaccines work by being translated by host ribosomes after injection, where the ribosomes will produce the protein that will serve as the disease antigen encoded by that mRNA transcript [100]. The mRNA vaccines are produced in vitro and have a much faster turnaround than cell-based vaccine production [101]. The success of mRNA vaccines produced in cell-free platforms demonstrates cell-free technologies’ utility over cell-based platforms, as well as its growing relevance in pharmaceutical manufacturing. Cell-free platforms also promise to deliver vaccines portably, specifically to developing nations where distribution is cost-prohibitive and hindered by storage requirements [93]. While other portable platforms, including chemistry-based systems [102] and microfluidic yeast bioreactors [103], provide additional solutions to this problem, both of these systems require special considerations that are unnecessary in a comparable cell-free platform [93]. These special considerations include experienced technicians and operating procedures and, in the example of yeast, international biosafety regulations associated with bringing live, engineered cells into foreign countries [93]. In contrast, demonstrated preconfigured cell-free systems would only require very basic protocols, such as the addition of water and incubation at room temperature, to begin the production of a targeted vaccine in a rural area [93]. The production scheme could also be expanded to include the production of a diverse repertoire of therapeutic biomolecules whose storage or production requirements currently make an efficient distribution to rural areas challenging [93].

#### 6.1.4. Small Molecules

Small molecules are powerful therapeutics that interact with a variety of different molecular processes within the cell. Small molecules mechanism of action usually involves inhibiting an endogenous molecule or binding site important to the function of a particular pathway to study or stop its function. An example of this is protein kinase inhibitors, which treat cancer by inhibiting signal transduction pathways that cancerous cells can utilize to metastasize [104]. Small molecules are also used to treat respiratory and autoimmune diseases and diabetes, demonstrating many potential applications in disease treatment [104]. The most powerful iteration of small molecule pharmaceuticals is arguably the aforementioned antibody–drug conjugates (ADCs), which combine small molecule and monoclonal antibody therapies [104]. Small molecules have traditionally been produced via cell lines. However, demand for highly potent active pharmaceutical ingredients (HPAPIs) requires new innovative production platforms. HPAPIs, as well as active pharmaceutical ingredients (APIs) generally, are small molecules that, with the increased specificity of novel treatments, have been found to be effective in oncological, autoimmune, and diabetes treatments [104]. HPAPIs are very toxic and therefore present challenges for manufacturing in cell-based systems that are sensitive to cytotoxic conditions [104]. Cell-free platforms would provide a potential solution to the challenge of producing toxic APIs because these platforms are resistant to toxic conditions and are not bound by cellular homeostatic concerns [19,105]. Although cell-free production of APIs is not well documented, in vitro platforms are well documented in the synthesis of biomolecules that are toxic and difficult to produce in cell-based platforms [106]. Apart from producing toxic APIs, cell-free small molecule manufacturing could also provide other advantages to the current cell-based production of small molecules, namely in production time and real-time observation and screening. One company actively developing this technology is Design Pharma, a biotechnology company utilizing CFPS to build and screen small molecules for use as pharmaceuticals [107].

#### 6.1.5. Membrane Proteins

Membrane proteins, while not pharmaceutical drugs themselves, are valuable targets for pharmacological research and development. Membrane proteins have a plethora of critical cellular functions, including signal transduction, small molecule transport through the cell membrane, cell surface and substrate binding, and reaction catalysis [108]. The many functions of membrane proteins make them prime targets for drug design, with approximately 60% of therapeutic drugs being designed to target membrane proteins [108]. Of that 60%, more than one-third of those therapeutics target the G protein–coupled receptor (GPCR) class of membrane proteins, vital in controlling signal transduction pathways [108]. Recently, new research in drug design for membrane protein targets focuses on the interactions that transmembrane domains (TMDs) make with each other [103]. These interactions are called protein-to-protein interactions (PPIs) and play an essential role in signal transduction [108]. In the past, these interactions were categorized under a group of regions thought to be “undruggable”; however, novel therapeutics with increased specificities are challenging this idea [108]. With new drug targets comes new opportunities to test the expression of these dynamic membrane proteins in cell-free systems. Membrane proteins have traditionally been produced in CHO cells; however, their status as difficult-to-express proteins makes production challenging [109]. The utilization of CHO lysates for CFPS has been demonstrated as a solution to many of these production challenges, optimizing the synthesis of membrane proteins that are often post-translationally modified [109]. Other cell-free platforms have also demonstrated efficient expression of many different membrane proteins and promise to become more widely used for membrane protein expression [110]. One such alternative system has been developed by the biotechnology company Synthelis, which utilizes an *E. coli*-based cell-free platform for the production of membrane proteins [111,112].

#### 6.1.6. On-Demand Production

Cell-free biomanufacturing is poised to continue to grow into the next decade, due in part to technological advancement allowing for the scalability of cell-free systems to industrial production levels. The growth is related to increasing demand for biomanufactured products such as pharmaceuticals [75,76,77]. Despite this increase, the distribution of critical pharmaceutical products such as vaccines, especially to low-populated areas, remains challenging [28,93]. Proper storage is the main challenge with distribution, as many pharmaceutical products have cold temperature requirements to remain viable. Compounding this problem is the fact that many niche pharmaceuticals that are vital for a small subset of people are incompatible with industrial production platforms because manufacturing them is cost-prohibitive [28]. A potential solution to both of these problems is to use a cell-free platform for on-demand production of target molecules, alleviating issues of proper distribution and allowing for more cost-effective production of niche medicines than what is possible in large volume formats [28]. As referenced earlier, there are competing chemistry and cell-based solutions for solving problems in the proper distribution of vaccines; however, they require complicated steps and on-sight expertise [93]. By contrast, an on-demand CFPS system would need only a minimal setup to begin protein synthesis [93]. Examples of on-demand CFPS systems have already been demonstrated in the literature for vaccines, VLPs, antimicrobial peptides, and antibodies, potentially opening a wider range of specialty therapeutics to areas previously difficult to reach [27,93,113]. Systems are also being developed to be highly portable and could see use beyond distribution to rural areas to point-of-care applications broadly [114,115]. Similar systems are also being developed that seek to automate the process of protein production as much as possible, demonstrating the synthesis of oligonucleotides from a digital copy of a DNA sequence to the subsequent transcription and translation of that sequence to functional target proteins in a completely automated format [116]. Companies such as Nuclera Nucleics, Liberum Bio, and Tierra Biosciences have also developed their own on-demand systems, suggesting that the use of on-demand systems for bioproduction will continue to grow [117,118,119]. Nuclera Nucleics is developing a desktop bioprinter with cartridge-based next-day gene and protein synthesis. This desktop bioprinter will integrate three advanced synthetic biology technologies: enzymatic DNA synthesis [120], digital microfluidic [121], and cell-free protein synthesis [122,123]. As a result of the development of the bioprinter, the operator will be able to take the DNA or protein sequence and next day harvest the synthesized protein. Challenges do remain in producing therapeutics on-demand, namely in producing functional glycoprotein products such as monoclonal antibodies using an on-demand cell-free system [124]; however, with continued research, cell-free platforms for the on-demand production of target molecules will continue to become more viable.

### 6.2. Food Biotechnology

Food biotechnology is generally defined as all processes encompassing the engineering of biological platforms to produce consumer food products. A natural ingredient, nutraceutical, and functional food production represent emerging subfields within food biotechnology that are gaining increasing popularity and market size [125]. The nutraceutical market was reported to be valued at nearly 200 billion dollars with a CAGR of 7.5% in 2016, while the functional foods market was reported to be valued at almost 65 billion dollars with a CAGR of 7.4% [125]. The large size and projected growth of the food biotechnology market highlight the potential opportunity for increased cell-free use in food biomanufacturing.

Presently, natural ingredients that are used in a variety of food productions are produced via cell-based bacterial and yeast production platforms [125]. Microbial fermentation systems suffer from a myriad of disadvantages, such as low yields and the inability to produce many complex ingredients found in nature [126,127]. As a result, many ingredients must be extracted from natural sources, namely from plants and insects, which is often expensive and time-consuming. Examples include the natural dye carmine, which is extracted from cochineal insects, and many carotenoids, which must be either extracted from plants or produced in inefficient chemical synthesis reactions [128,129]. Cell-based systems also present additional difficulties in production, such as the time-consuming process of reprogramming cells to produce new molecules and the use of batch processing which limits reaction time and resource availability [37,38,126]. These limitations are beginning to make cell-free systems more attractive to ingredient manufacturers, such as Debut Biotech, a biotechnology company utilizing cell-free systems to produce natural food ingredients without the limitations associated with standard fermentation [130]. Cell-free platforms benefit from a continuous production process allowing for the constant influx and use of resources to prolong reaction times and increase yields, unlike the limited batch formats used for cell-based systems [37,38]. The continuous format also allows for smaller infrastructure requirements than batch systems, which require large fermenters to produce functional products [37,38]. Cell-free systems are also much more resilient to changes in pH than cell-based systems, which is an important advantage for the production of food ingredients [46,126]. Food ingredients are produced naturally in organisms at variable pHs; however, cell-based systems must be kept at neutral pH to maintain homeostasis. This results in products that are different in quality from their natural counterparts. Using cell-free systems, however, the natural conditions of ingredient production can be mimicked to a much higher degree, resulting in a product that more closely resembles those found in nature [46,126]. The advantages of cell-free allow for a larger range of potential ingredients to be produced than is possible in cell-based systems and will lower the cost and time spent either producing these ingredients or extracting them from natural sources [46,126]. Ingredients include probiotics, prebiotics, proteins, amino acids, dietary fibers, and vitamins.

### 6.3. Growing Industries & Industrially Relevant Biomolecules

Besides the pharmaceutical and food industries, other industries such as cosmetics and industrial biomolecule production can benefit from cell-free systems. The global cosmetic industry is growing steadily with a CAGR of 4.3%, where it is expected to be valued at 450 billion dollars by 2025 [131]. The cosmetic industry utilizes a diverse range of biomolecules, one category of which is fatty acids. Fatty acids are typically produced in cell-based systems, but increasing demand for fatty acids in cosmetics, nutritional supplements, and as a biofuel is driving production to cell-free systems [132]. Many cosmetics also contain proteins, with factors such as solubility and hydrophobicity determining what types of cosmetics they are used in [133]. For instance, insoluble proteins such as fibrous collagen are popular ingredients in face masks, while proteins with high molecular weights such as the collagen derivative desamidacollagen are used in skincare products [133]. Collagen and its derivatives are well-documented in CFPS experiments, and cell-free platforms could be optimized to produce collagen for cosmetic applications [134].

Also growing is the industrial enzymes market, which was projected to be worth over 7 billion dollars in a 2018 report, up from 4.2 billion dollars in 2014, with a CAGR of 8.2% [135,136]; along with the biopolymers market, which is currently worth more than 3.6 billion dollars with a CAGR of 14.5%, signifying the demand for biopolymers in a variety of different industries [135]. Popular industrial enzymes for biopolymer production include laccase and cellulose, produced in *Bacillus subtilis* bacterium [135]. Although the cell-free production of these enzymes and other enzymes involved in biopolymer production is not well elucidated in the literature, Bacillus subtilis based cell-free systems have been created [137], and cell-free systems could conceivably be optimized to produce these enzymes.

## 7. Utilizing Machine Learning in Cell-Free

Machine learning (ML) is a type of artificial intelligence that uses data analysis to improve modeling methods for a variety of different applications such as speech pattern recognition, autonomous vehicles, and finance. In the last decade, there has been a shift towards data-driven modeling rather than physical modeling, in particular concerning biochemical processes, which are one of the most complex systems in the real world. The most used machine learning methods comprise artificial neural networks, ensemble learning, multivariate statistical analysis, and Gaussian processes. Models are built for knowledge discovery, to predict system outcomes, and to save time. However, to reduce the possible uncertainty of machine learning processes, it is necessary to build novel physical models which improve human understanding of the underlying system [138].

ML has been utilized in several biological applications, such as drug discovery, to validate targets of therapeutics [139] as well as in genomics to assist geneticists in ordering large and complex datasets [140]. Machine learning methods can be applied to CFPS, an open system composed of many components underlying a multidimensional experimental space in which concentration levels can be independently adjusted to find an optimal configuration.

**Figure 3:**
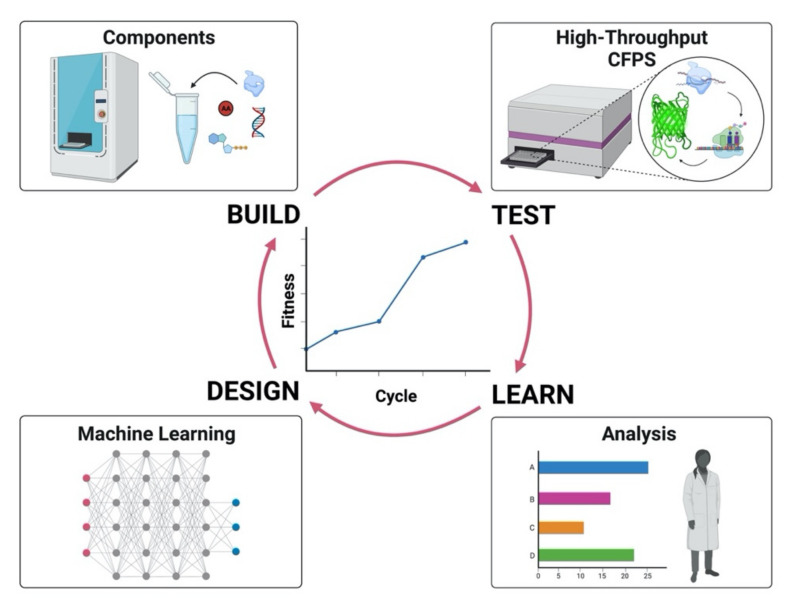
Design-Build-Test-Learn cycle involving machine learning. The figure illustrates the iterative cycle, comprising four steps, towards an improved system applying machine learning.

Cell-free transcription/translation systems are influenced by a complex set of factors interacting nonlinearly and synergistically. The first example of a CFPS system optimized by a machine learning algorithm was a robotic workstation coupled to high-throughput experiments [29]. This approach corresponds to an evolutionary design of experiments (Evo-DOE), where the initial human input that defines the experimental space is followed by a machine learning algorithm based on an artificial neural network (ANN) that predicts the next round of experiments towards improvements of the fitness function. With this method, an increase in the yield of a fluorescent reporter protein and synergies among important components of the energy buffer were demonstrated. The experimental space was composed of more than one million possible combinations in 16-dimensional space, and exploring only a small subset of combinations (0.014% of the total), yielded a threefold increase. Furthermore, the same machine learning method was applied to optimize more complex CFPS systems for ribosome construction, i.e., iSAT (in vitro integrated synthesis, assembly, and translation), likewise changing the concentration of the components in the energy buffer [141]. The complexity of the system arises from the complication of setting up an automated protocol for making active ribosome in vitro, from exploring a 20-dimensional space that gives a total of ~ 1.1 × 10^12^ possible combinations, as well as from the robotic constraints which allowed only the exploration of a pivot experiment in the space and neighborhood points. However, despite the limitation imposed by the robotic workstation, the machine learning algorithm could increase the yield of synthesized protein tenfold, discover significant intercomponent synergies, and decrease the cost of the cell-free reaction 4-fold by testing only 553 different combinations over a trillion possible recipes. Figure 3 shows the design-build-test-learn (DBTL) cycle applied to optimize cell-free systems using ML algorithms. The machine learning algorithm [29,141] developed to improve CFPS is available on the cloud at the following website: https://daptics.ai/ accessed on 2 December 2021.

Another example of a machine learning algorithm based on ANN was applied to improve the yield of a reporter GFP in an 11-dimensional experimental space that gave a total of 4 million possible combinations of the components of the energy buffer. The authors achieved a 34-fold increase in protein yield [142]. The methods described above are defined as active learning and involve the use of multiple machine learning algorithms to design future experiments during the study of a single problem [143]. Interestingly, a data-driven approach based on ANN was also used to optimize the construction of a metabolic pathway in vitro using CFPS. The results showed a strong correlation of the in vitro optimized pathways with its expression in vivo. In particular, a 20-fold improvement of the production of 3-HB in vivo was observed compared to a basal case [47]. Overall, these studies show the power of machine learning and high-throughput screening in exploring large and complex experimental spaces, specifically in optimizing metabolic pathways that are prototyped as well as improving the yields of protein produced in vitro. However, concerning the optimization of CFPS yields, it must be mentioned that selected parameters could be specific for the target protein, and this represents a form of overfitting that can be solved only by performing more experiments using other target proteins [141]. Concerning the optimization of CFPS yields in a multidimensional experimental space, there are also examples where the multivariate statistical analysis was applied, requiring less computational resources [144,145,146]. For instance, multivariate statistical data analysis (MSDA) was applied to predict the yield of monoclonal antibodies (MAbs) using a scalable cell-free expression system developed by Sutro Biopharma [144]. In particular, process parameters such as pH, temperature, and O_2_ affect yield and aggregation. Applying this method, the authors showed an accurate prediction of end-point product quality [144]. Interestingly, MSDA was applied to optimize the yields of insect-based cell-free expression systems changing the composition of the translation premix, which energized the system. Based on the model prediction, optimal components concentrations could be validated for the insect lysate [146]. This optimized eukaryotic cell-free expression system is interesting because of the many possible protein post-translational modifications using this type of lysate.

CFPS systems are multidimensional problems with complex fitness landscapes. Optimization approaches based on intuitions of components’ interdependence, such as the One Factor at A Time (OFAT) method, involve measurements of the system output while each variable is changed in turn and other parameters are held constant. This is time and resource-intensive and ignores nonlinear interactions, a common characteristic of biological systems [147]. Therefore, to overcome these limitations, statistical methods based on data-driven design are more suitable in optimization processes. Typically, the screening design of large complex experimental spaces identifies a small subset of parameters that greatly influence the system’s fitness. Subsequently, experimental budgets are allocated to explore their effect and hold constant or remove those variables that are statistically irrelevant [147]. Overall, the iterative experimentation based on statistical modeling of the fitness landscape reduces the need for a priori knowledge of the system based on biophysical or mechanistic models [147]. As a result, in the context of cell-free expression system optimization, active learning is perfectly suitable because of the large amount of data generated quickly in cell-free formats, allowing for a significant degree of optimization by machine learning algorithms in shorter time spans than what would be possible with noncell-free systems [142]. Indeed, cell-free expression systems are moving towards workflow standardization and automation [122] that perfectly support efficient system design by machine learning-based experimentation. In conclusion, ML and automation generally show great promise in streamlining experimental and industrial processes, particularly cell-free systems.

## 8. Conclusions

Cell-free systems have undergone many iterative improvements since their inception over a century ago. From simple cell lysates that proved that basic molecular processes such as fermentation could occur independently of living cells, to industrially optimized platforms capable of producing a diverse range of target molecules, cell-free systems are well poised to become the next-generation platform for a range of applications in biotechnology. Current cell-based systems, while themselves iterative improvements over previous chemical synthesis platforms, are ill-equipped to handle the growing fields of metabolic engineering and biomanufacturing, where subfields such as drug discovery and biosensor development will require a more diverse range of reaction conditions and target molecules than they can supply. Conversely, cell-free production continues to improve, both technologically, in the preparation of extracts and the development of purified component platforms, as well as economically, with the use of low-cost reagents and efficient production cycles. Cell-free advantages in reaction condition tolerance and customization make the platform ideal for prototyping new metabolic pathways and genetic circuits, and further use of cell-free systems will result in more efficient experimental and industrial processes. These advantages also make the platform attractive for the industrial production of biomolecules, whether pharmaceuticals, food products, or cosmetics.

Production hurdles such as resource exhaustion and limited yields will continue to diminish as new methods of preparing extracts, new methods of energy regeneration, and larger batch volumes are achieved. Cell-free platforms will also greatly benefit the niche and hard-to-distribute sectors of the pharmaceutical market, producing critical therapeutics on-demand with little to no infrastructure, bypassing the storage requirements of traditional distribution, and opening up the production of a whole host of therapeutics deemed cost-prohibitive with current production schemes. Aiding these cell-free applications will be artificial intelligence, specifically machine learning and automation, increasing production yields through improving reaction conditions and lysate components, as well as utilizing active learning algorithms to design new experiments tackling future roadblocks. Cell-free platforms are not perfect, extract-based systems still suffer from side reactions during energy metabolism that limit efficiency, and enzyme-based systems still suffer from the cost-prohibitive aspects of scalability; however, just a decade ago, many of these platforms’s current abilities were deemed impossible, and the systems continue to improve at a rapid pace. Cell-free systems have proven they are adaptable to a diverse range of applications, and the next generation of cell-free researchers will continue to build on the progress that has been made, expanding the horizons of what is possible outside of the cell.
